# The effect of capture and handling stress in *Lophius americanus* in the scallop dredge fishery

**DOI:** 10.1093/conphys/coy058

**Published:** 2018-10-26

**Authors:** Amelia M Weissman, John W Mandelman, David B Rudders, James A Sulikowski

**Affiliations:** 1 Marine Science Center, University of New England, 11 Hills Beach Rd., Biddeford, ME, USA; 2 Anderson Cabot Center for Ocean Life, New England Aquarium, 1 Central Wharf, Boston, MA, USA; 3 Virginia Institute of Marine Science, College of William and Mary, 1375 Greate Rd., Gloucester Point, VA, USA

**Keywords:** bycatch, fisheries, monkfish

## Abstract

Capture and handling stress studies are considered a primary research priority, particularly for species and fisheries where discard rates are high, and/or for overfished stocks and species of concern. *Lophius americanus*, a commercially valuable finfish in New England, constitutes the second highest bycatch species within the sea scallop dredge fishery. Despite its commercial importance, no data exists on the capture and handling stress of monkfish for any gear type. Given these shortcomings, our goals were to evaluate the stress response of monkfish captured in scallop dredge gear by evaluating physical, behavioural and physiological responses to scallop fishing practices. While 80% of monkfish displayed little to no physical trauma, behavioural and physiological assessment indicated high levels of stress, especially as air exposure and tow duration increased. This finding suggests that the manifestation of stress in monkfish may be a cryptic response necessitating further research in addition to estimates of post-release mortality rates to appropriately advise fisheries management regarding the mortality of monkfish bycatch in the sea scallop fishery.

## Teaser

The objective of this study was to determine the organismal stress response using physical, behavioural and physiological indicators in monkfish captured in the scallop fishery. While most fish displayed little to no injury, behavioural and physiological impairment indicated high levels of stress associated with capture and handling practices.

## Introduction

Bycatch is defined by Alverson *et al.* (1994) as any non-target species captured at sea while discarded catch is described as ‘that portion of the catch returned to the sea as a result of economic, legal, or personal considerations.’ For the purposes of this paper, the term bycatch is used to describe the catch of non-target species, whether retained or discarded. The phenomenon of bycatch, while broadly defined, affects all fisheries regardless of gear type (Magnuson Stevens Act, 1996; Crowder and Murawski, 1998; Kirby and Ward, 2014). Since its implementation in 1996, the Magnuson Stevens Act of the USA has encouraged a variety of practices including gear modification, catch limits and area closures to minimize the bycatch of marine species and thus the need to discard any catch (Magnuson Stevens Act, 1996; Kirby and Ward, 2014; O’Keefe *et al.*, 2014). While these efforts have, to varying degrees, mitigated bycatch for some species (O’Keefe *et al.*, 2014), bycatch remains a major problem for fisheries primarily due to the negative impacts of the stress of capture and handling on bycaught organisms which can lead to subsequent mortality after discard (Davis, 2002; Danylchuk *et al.*, 2014; Uhlmann *et al.*, 2015; Schlenker *et al.*, 2016).

Capture and handling stress are two of the most challenging factors to characterize when studying bycaught organisms (Davis, 2002). This is largely due to the potentially broad array of interactive factors, such as physical injury, rapid temperature and pressure changes and exposure to air, which can act as stressors (Davis, 2002; Baker *et al.*, 2013). Recent studies have attempted to quantify the stress response associated with the capture and handling process. For example, physical responses such as external injury and reflex impairment have been utilized to assess the stress of capture in teleost species including yellowtail flounder (*Limanda ferruginea*) (Barkley and Cadrin, 2012), Atlantic cod (*Gadus morhua*) (Humborstad *et al.*, 2016) and bluegill (*Lepomis macrochirus*) (Lennox *et al.*, 2015). In addition to utilizing overt trauma as predictors of mortality, physiological responses to the capture and handling process have been quantified via changes in such biochemical markers as cortisol, lactate, glucose and mean corpuscular haemoglobin content (MCHC) that can aid in quantifying stress (Donaldson *et al.*, 2013; Uhlmann *et al.*, 2015; Barkley *et al.*, 2017). These responses, however, can be nuanced which increases the complexity of understanding this issue. Since the stress response cannot be generalized across taxa, it is important to study this effect as a function of fisheries-related stressors at the species and gear level (Barton, 2002). The physiological and physical effects due to capture and handling may lead to delayed mortality, which can have direct ecosystem impacts, but also influences fisheries management (Davis, 2002; Benoit *et al.*, 2015). Bycatch species composition and associated discard mortality rates directly affect target catch quotas in many fisheries (Crowder and Murawski, 1998; Benoit *et al.*, 2015). Therefore, it is crucial for fisheries managers to have accurate information regarding the effect of fishing gear type on bycaught species, so that appropriate management decisions can be made (Barkley and Cadrin, 2012; NEFSC, 2012).

One of New England’s most commercially valuable finfish is the monkfish, *Lophius americanus*, with a directed fishery worth over $19 million ex-vessel in 2014 (Lowther and Liddel, 2015). In addition to the directed fishery, monkfish also represent 13% of the total bycatch for the sea scallop dredge fishery (NMFS, 2011). Despite the economic importance and high bycatch rate, there have been no directed studies that investigate the effect of sea scallop dredge fishing on the health and survival of *L. americanus.* Given the paucity of relevant information, the objectives of this study were (1) to test four vitality reflexes to characterize sub-lethal effects as a function of fishing stressors, (2) to evaluate injury condition as a predictor of subsequent mortality and (3) to measure plasma cortisol, and whole-blood haemoglobin, haematocrit and lactate levels to assess the physiological status of capture and handling stress associated with capture in commercial scallop gear.

## Materials and methods

### Animal collection

*L. americanus* were opportunistically sampled during four, week-long sea scallop dredge cruises dedicated to sea scallop research between June and December 2015. The fishing gear utilized was a standard New Bedford style sea scallop dredge which was equipped with a steel cutting bar and sweep chain that dredge the benthos and collect organisms or debris in the ring bag as the gear is towed behind the vessel (Yochum and DuPaul, 2008). The June cruise occurred on Georges Bank, while the other three cruises (July, August and December), occurred along the Mid-Atlantic Bight. Individual dredge tow durations were randomly assigned to last between 10 and 90 min (the approximate range of tow duration typical for the fishery), with a 5-min tow occurring every 20 tows to serve as a minimally stressed reference group. Otherwise, fishing reflected normal industry practices, operating both day and night (Yochum and DuPaul, 2008). At the beginning of each cruise, temperature loggers (Hobo Water Temp Pro v2, Onset Computer Corporation, Bourne, MA) were placed on deck as well as attached to the dredge to record air and bottom water temperatures, respectively. After the dredge was deployed and had been towed for the predetermined duration, geographic coordinates and water depth were recorded. To quantify air exposure, a stopwatch was started once the gear was out of the water and air exposure durations per sampled fish were recorded when assessment began. The contents of the dredge were emptied on deck and the fishermen sorted through the catch, removing all retained scallops. After the scallop catch was removed and only bycatch remained on deck, captured monkfish were randomly selected for evaluation. All retained fish were measured for total and precaudal length, assessed for injury and reflex impairment and attempted to have a blood sample drawn.

### Injury condition and reflex responses

As a method of assessing vitality, each captured fish was assigned an injury condition and tested for reflex responses (Barkley and Cadrin, 2012; Humborstad *et al.*, 2016). Injury condition and reflex responses were analyzed separately to distinguish the effects of physical trauma from behavioural impairment. These responses were based on reflexes developed for other species that were modified to apply to monkfish. The efficacy of the derived reflexes was evaluated via pilot studies on the species. The monkfish vitality index was comprised of a combination of an ordinal injury score (1 = uninjured; 2 = minor damage; 3 = severe trauma; 4 = dead) and four reflex responses: (1) mouth (probe insertion to assess jaw closure), (2) eye fixation (rotation of fish to observe pupil fixation), (3) back arch (fish placed on dorsal side to observe spinal arch) and (4) thrash (body flex stimulated by handling).

### Physiological analysis

To evaluate changes in physiological state, 3 ml of blood was collected from each sampled fish via the caudal vein using a heparinized syringe with a 22-gauge needle after injury condition was assessed and reflexes were tested. Three fish representing both injuries 1 and 2 from short to medium tow durations and air exposure times were used as minimally stressed reference groups. Lactate (Lactate Plus, Nova Biomedical, Waltham, MA), glucose (Nova Max Plus, Nova Biomedical, Waltham, MA), and haemoglobin (Hemocue HB 201+, HemoCue America, Brea, CA) concentrations were determined using handheld meters shown to be effective with other teleost species (Rummer *et al.*, 2013; Collins *et al.*, 2016). However, since all values were lower than the range of the handheld meter (<20 mg/dl), glucose concentrations could not be determined. Haematocrit (packed erythrocyte volume percentage) was measured through standard techniques (Sulikowski *et al.*, 2003) and mean corpuscular haemoglobin content (MCHC) was determined from the haemoglobin to haematocrit ratio (Sulikowski *et al.*, 2003). The remainder of the blood was stored in heparinized vacutainers and refrigerated overnight to allow the plasma to separate from the red blood cells. The plasma was removed and stored frozen for further analysis.

At the University of New England, cortisol concentrations were determined using a standard radioimmunoassay (RIA) technique modified from Tsang and Callard’s (1987) protocol and Sulikowski *et al.* (2004). Each plasma sample was spiked with 1000 counts min^−1^ of tritiated cortisol (Perkin Elmer, Waltham, MA) and extracted twice with 5 ml of ethyl ether (ACS grade). The extracted samples were evaporated under nitrogen and each was reconstituted in phosphate-buffered saline with 0.1% gelatin. The mean extraction recovery value was calculated as 70.4%. For the RIA, non-radiolabeled hormones were obtained from Steraloids, Inc. (Newport, RI) to make a stock solution at 100 μg ml^−1^ in 100% 200-proof ethanol (ACS grade). Radiolabeled hormones were obtained from Perkin Elmer (Waltham, MA), and antibodies from Fitzgerald Industries (Acton, MA). Radioactivity was detected using a Perkin Elmer Tri-Carb 2900TR liquid scintillation analyzer (Waltham, MA). Inter-assay and mean intra-assay coefficients were calculated and reported to be 23.9% and 8.0%, respectively.

### Statistical analysis

A chi-square goodness of fit test was performed to determine if injury condition was significantly different between the two study sites. A G-test was performed to determine if there was a significant difference in the average number of reflex responses among each injury condition (Gotelli and Ellison, 2013). Logistic regressions were performed to determine which technical, biological, and environmental factors (e.g. month, air exposure duration, tow duration, depth of capture, total length and temperature difference) were significant predictors of both injury condition and reflex response. Linear regressions also were performed to determine which technical, biological and environmental factors (e.g. month, air exposure duration, tow duration, depth of capture, total length and temperature difference) influenced physiological status analyzed from the blood samples. Generalized additive models were performed to determine any significant interactive effects of technical, biological and environmental factors (e.g. month, air exposure duration, tow duration, depth of capture, total length and temperature difference) on injury condition, reflex responses and each physiological parameter. If the data were not normally distributed or displayed heterogeneity of variance, then the data were log transformed and statistical outliers (determined with Bonferroni outlier test) were removed. Table 1 displays all the models utilized and their respective variables. Statistical significance was accepted if *P* < 0.05. All statistical tests were performed in R 3.2.5 (The R Foundation).

**Table 1:** Statistical analyses utilized for the current study with their respective explanatory and response variables

Statistical test	Explanatory variable(s)	Response variable	Comments
Chi-square	Location	Injury Condition	
G-test	Injury Condition	Reflex Responses	
Logistic Regression	Exposure Time	Injury Condition	Log-transformed
Logistic Regression	Tow Duration	Injury Condition	
Logistic Regression	Exposure Time	Reflex Responses	Log-transformed
Logistic Regression	Tow Duration	Reflex Responses	
Linear Regression	Exposure Time	Lactate Concentration	Log-transformed, 3 outliers removed
Linear Regression	Tow Duration	Lactate Concentration	Log-transformed, 2 outliers removed
Linear Regression	Exposure Time	MCHC	1 outlier removed
Linear Regression	Tow Duration	MCHC	Log-transformed
Linear Regression	Exposure Time	Cortisol Concentration	Log-transformed
Linear Regression	Tow Duration	Cortisol Concentration	Log-transformed
Generalized Additive Model	Month × Exposure Time × Tow Duration × Depth × Temperature Difference × Total Length	Injury Condition	
Generalized Additive Model	Injury Condition × Month × Exposure Time × Tow Duration × Depth × Temperature Difference × Total Length	Reflex Responses	
Generalized Additive Model	Injury Condition × Month × Exposure Time × Tow Duration × Depth × Temperature Difference x Total Length	Lactate Concentration	
Generalized Additive Model	Injury Condition × Month × Exposure Time × Tow Duration × Depth × Temperature Difference × Total Length	MCHC	
Generalized Additive Model	Injury Condition × Month × Exposure Time × Tow Duration × Depth × Temperature Difference × Total Length	Cortisol Concentration	

## Results

**Figure 1:**
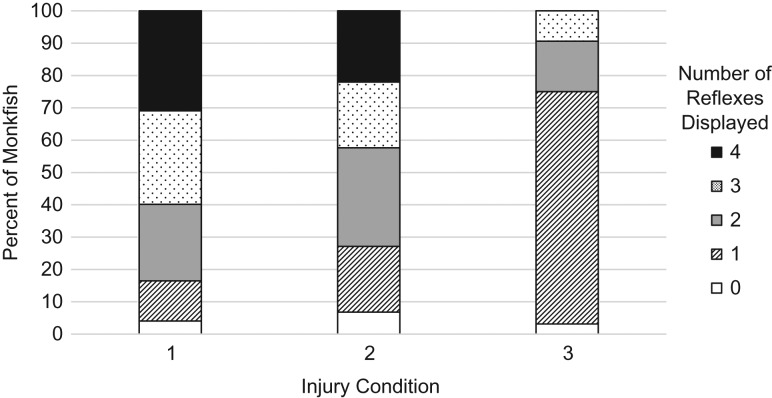
Reflex responses as a function of injury condition for *L. americanus* captured in commercial scallop dredge gear. The number of reflex responses were significantly reduced as injury condition increased (worsened) (G-test; *χ*^2^ = 59.58, *P* < 0.0001).

A total of 483 monkfish (ranging in total length from 10.2 to 88.0 cm) were assessed for injury condition over the course of the four cruises. There was a significantly higher percent of injury 4 (dead) fish (*χ*^2^ = 11.81, *P* = 0.008, Table 2) in the Mid-Atlantic cruises compared to the Georges Bank cruise. There was a significant difference in the number of reflexes present among injury conditions (*χ*^2^ = 59.58, *P* < 0.0001; Fig. 1), however this difference was driven by the high percent of one reflex present in injury three fish. The number of reflexes present for fish coded injury 1 or 2 displayed similar proportions (Fig. 1).

**Table 2:** Sample size of *L. americanus* representing each injury code captured in the scallop dredge fishery between both study sites on Georges Bank and in the Mid-Atlantic Bight from June to December 2015

	% Injury 1 (*n*)	% Injury 2 (*n*)	% Injury 3 (*n*)	% Injury 4 (*n*)
Georges Bank	66.7% (74)	20.7% (23)	9.9% (11)	2.7% (3)
Mid-Atlantic Bight	61.8% (230)	15.6% (58)	8.1% (30)	14.5% (53)

**Figure 2:**
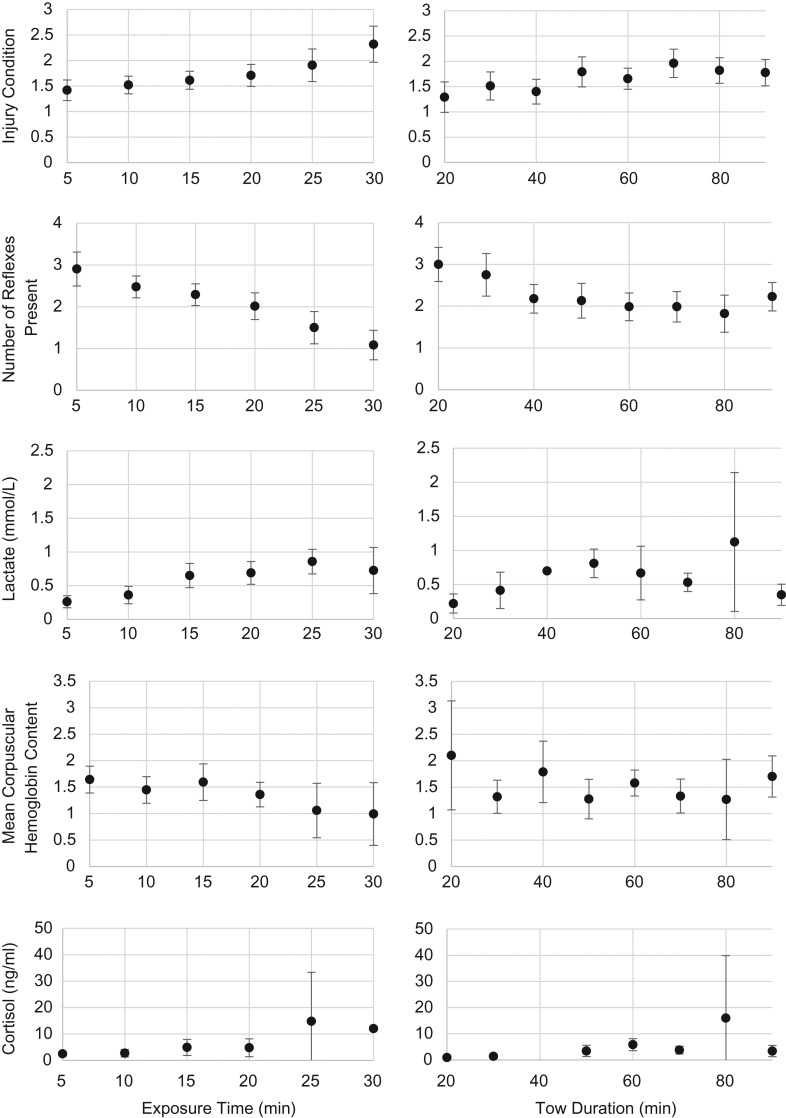
Results of physical and physiological responses of *L. americanus* to capture and handling stress represented by tow duration and air exposure duration, respectively. Results of the injury condition and reflex responses generated from logistic regression analyses. Results of the lactate concentrations, MCHC, and plasma cortisol concentrations generated from linear regression analyses. Data are mean physical or physiological response binned by 5 min exposure time intervals and 10 min tow intervals. Error bars represent 95% confidence intervals

The results from the logistic regression analyses reveal that monkfish displayed significant increases in injury condition (Table 3; Fig. 2), and significant decreases in number of reflexes present (Table 4; Fig. 2) as air exposure time and tow duration increased, respectively. Linear regression analyses determined that a significant increase in lactate concentrations (*F *= 39.83, *P* < 0.0001, Fig. 2) and significant decrease in MCHC (*F* = 7.52, *P* = 0.007, Fig. 2) was observed as air exposure time increased. However, there was no significant change in lactate concentrations (*F* = 2.52, *P* = 0.116, Fig. 2) or MCHC (*F* = 0.31, *P* = 0.576, Fig. 2) as tow duration increased. Plasma cortisol concentrations significantly increased (*F* = 9.73, *P* = 0.003; *F* = 18.98, *P* = 0.0001; Fig. 2) as air exposure and tow duration increased, respectively. Air exposure durations ranged from 3 to 38 min. Ranges and averages of additional independent variables which did not yield significant results are described in Table 5.

**Table 3:** Statistical results of the logistic regression models for the effect of tow duration and air exposure duration on injury condition of monkfish captured in the scallop dredge fishery from June to December 2015

Injury condition	Tow duration		Air exposure duration	
	*Coefficient*	*P-value*	*Coefficient*	*P-value*
2	−1.804	0.012	−1.491	<0.001
3	−3.060		−3.573	
4	−2.787		−2.925

**Table 4:** Statistical results of the logistic regression models for the effect of tow duration and air exposure duration on reflex responses of monkfish captured in the scallop dredge fishery from June to December 2015

Reflex responses	Tow duration		Air exposure duration	
	*Coefficient*	*P-value*	*Coefficient*	*P-value*
1	−0.440	0.007	0.609	<0.001
2	0.807		0.665	
3	1.334		2.079	
4	0.994		2.539

**Table 5:** Ranges and averages of non-significant environmental independent variables recorded during scallop dredge cruises from June to December 2015. Temperature differential was calculated by subtracting the bottom water temperature from the air temperature

	Depth (m)	Air temperature (°C)	Bottom water temperature (°C)	Temperature differential (°C)
Average	56.1	23.3	6.6	15.5
Range	40.2–76.8	8.7–35.6	5.1–8.5	−9.1–27.5

The results of the generalized additive models yielded interactive effects on physical, behavioural, and physiological impairment mainly driven by air exposure, month, and injury condition (Table 6). However, for the purposes of this study, we will only address air exposure and tow duration as separate stress-contributing factors in the discussion because these are the variables which contributed most to the stress responses and can be easily controlled by fishing practices.

**Table 6:** Statistical results of generalized additive models for the effect of interactive explanatory variables on the physical, behavioural and physiological state of monkfish captured in the scallop dredge fishery from June to December 2015. Asterisks indicated significance, while NS indicates no significance

Explanatory Variable	Injury Condition (CV = 60.692, SE = 0.052)	Reflex Response (CV = 64.719, SE = 0.077)	Lactate (CV = 173.463, SE = 0.163)	MCHC (CV = 54.410, SE = 0.089)	Cortisol (CV = 176.781, SE = 1.439)
Injury Condition	NA	***F* = 14.203**	***F *= 2.430**	Not significant	***F *= 2.136**
		***P* < 0.001**	***P* = 0.018**		***P* = 0.040**
Month	***F* = 8.127**	***F *= 14.203**	***F* = 2.175**	***F* = 14.301**	Not significant
	***P* < 0.001**	***P* < 0.001**	***P* = 0.034**	***P* < 0.001**	
Exposure Time	***F *= 6.389**	***F *= 15.38**	***F* = 5.764**	***F* = 6.244**	***F* = 7.401**
	***P* = 0.006**	***P* < 0.001**	***P* < 0.001**	***P* = 0.015**	***P* = 0.010**
Tow Duration	***F *= 1.901**	Not significant	Not significant	***F* = 2.428**	Not significant
	***P* = 0.045**			***P* = 0.019**	
Depth	***F *= 2.205**	Not significant	Not significant	Not significant	Not significant
	***P* = 0.004**				
Total Length	Not significant	Not significant	Not significant	Not significant	Not significant
Temperature Difference	Not significant	Not significant	Not significant	***F* = 3.604**	Not significant
				***P* = 0.031**	

## Discussion

Despite the economic importance of the sea scallop fishery and frequent capture of non-target species, information surrounding the disposition of fish captured by scallop dredge gear remains scant. In the present study, the majority (~80%) of monkfish displayed little or no outward physical injury following capture by scallop gear. This result differs from the only other study, conducted on several skate species, to directly evaluate the reflex impairment and injury from capture in this gear type. For example, Knotek *et al.* (2018), using a similar injury scoring system, observed injury rates of 78%, 81% and 89% among little (*Leucoraja erinacea*), winter (*L. ocellata*) and barndoor skates (*Dipturus laevis*) captured in scallop dredge gear, respectively. While direct comparisons are limited, similar studies conducted using other mobile gear can also offer insight into the degree of physical injury. For example, studies on species captured in otter trawl gear observed that 60% of skates (Benoit *et al.*, 2010) and 80% of groundfish (Mandelman *et al.*, 2013) species displayed significant physical injury, such as extruded intestines and major bleeding or tearing, from capture. Based on these comparisons, monkfish appear to be more resilient than other benthic species to physical stressors that can affect individuals upon capture. While the assessment of physical trauma suggested resilience, only 29% of the monkfish that were assessed as injury code 1 or 2 displayed all four reflex responses. Interestingly, these results contrast with work that has demonstrated physical injury correlates with reflex impairment (e.g. Davis, 2010; Barkley and Cadrin, 2012; Knotek *et al.*, 2018) and suggests a more cryptic stress response in monkfish. Since outward physical injury alone is not representative of the monkfish in the current study’s overall condition, biochemical markers (e.g. Sopinka *et al.*, 2016) were assessed to better understand the stress response of monkfish captured in scallop gear.

In the current study, capture in scallop gear produced elevated plasma cortisol levels in monkfish when compared to minimally stressed reference concentrations. For example, plasma cortisol values for monkfish with injury codes 1 and 2 were tenfold higher than plasma cortisol values in minimally stressed reference fish, and those assessed with an injury code 3 displayed plasma cortisol concentrations 100 times higher than minimally stressed reference values. Plasma cortisol concentrations also were observed to be significantly higher when air exposure exceeded 20 min and tow duration exceeded 70 min (Fig. 2), suggesting that monkfish may have a maximum threshold at which they can endure capture and handling stresses before they become physiologically compromised.

While direct comparisons of cortisol concentrations to other species is difficult given the variability among species, gear type, and methods (Barton, 2002), other benthic fish have demonstrated similar trends to those observed in the current study. For example, sea raven (*Hemitripterus americanus*) plasma cortisol concentrations increased five-fold (Vijayan and Moon, 1994) while wolffish (*Anarhichas minor*) plasma cortisol values increased fourteen-fold (Lays *et al.*, 2009) after a stressor was applied. In addition, plasma cortisol concentrations for benthic teleosts with low metabolic lifestyles, like monkfish, may peak more than 30 min post-stressor. For example, Vijayan and Moon (1994) and Lays *et al.* (2009) found that sea raven and wolffish, respectively, displayed elevated plasma cortisol 4 h post-stress, whereas more active species’, such as Eurasian perch and rainbow trout, plasma cortisol levels peaked within 30 min post-stress (Jentoft *et al.*, 2005). While average plasma cortisol concentrations in monkfish increased eighteen-fold in the current study, blood samples were restricted to only a maximum of 30 min post-stress and may not reflect peak plasma cortisol concentrations. Future research should temporally analyze blood samples collected at longer time intervals post-stress to determine if monkfish also possess a delayed response which could indicate a more severe physiological response than documented in the current study.

In addition to plasma cortisol, the current study also observed a significant increase in a secondary stress parameter, lactate concentration, and a significant decrease in MCHC as air exposure increased. These observations suggest, as in other species, that when air exposure increases, monkfish rely on anaerobic respiration resulting in lactic acid build-up and cell shrinkage due to lack of oxygen exchange (Gingerich *et al.*, 2007; Bhatkar, 2011; Ghaffar *et al.*, 2015; Barkley *et al.*, 2017). Heard *et al.* (2014) noted similar trends in stingrays captured in trawl gear, as the effects of crowding and compaction combined with extended trawl durations (up to 3 h) and air exposure (up to 1 h) resulted in significant increases in lactate concentration when compared to control values (Heard *et al.*, 2014). In contrast, glucose concentrations were too low to be detected in the sampled monkfish. A probable explanation for this may be related to the metabolic cost for coping with stress (Jentoft *et al.*, 2005). In this scenario, monkfish would be rapidly consuming glucose to maintain homoeostasis (Martinez-Porchas *et al.*, 2009). Other studies, such as those performed on rainbow trout and Eurasian perch, also demonstrate that glucose can remain low post-stress due to mobilization of energy reserves (Jentoft *et al.*, 2005). However, the blood glucose results should be interpreted with care, as extrinsic factors such as diet, time since last feeding, and season of the year, etc. may affect liver glycogen stores and thus circulating plasma glucose levels (Barton, 2002).

Overall, the results of this study suggest that *L. americanus* experience low physical injury, but the cumulative effects of capture and handling stress may be cryptic. This assertion is highlighted when considering observed behavioural and physiological stress. Collectively, the primary and secondary physiological responses, in addition to behaviour impairment, demonstrated by monkfish in the current study suggest this species is negatively impacted by capture and handling in the scallop dredge gear, especially as tow duration and air exposure increase. These findings demonstrate that injury alone does not accurately represent the true nature of stress incurred by capture and handling and must be analyzed in combination with behavioural and/or physiological indicators.

Results for this study suggest that consideration of operational factors could have an impact on the disposition of monkfish captured as bycatch in the sea scallop dredge fishery. With respect to one of the operational variables assessed, tow duration is likely a difficult variable to control in practice. With no current method of estimating how long fish are entrained in and affected by the gear, tow duration is a difficult variable to quantify and ultimately control from a management perspective. However, air exposure is much easier to assess, and by enacting best handling practices that encourage fishermen to return monkfish to the ocean immediately after capture may reduce the probability of post-release mortality due to capture and handling stress.
